# Expression profiles of sugarcane under drought conditions: Variation in gene regulation

**DOI:** 10.1590/S1415-475738420140288

**Published:** 2015

**Authors:** Júlio César Farias de Andrade, Jackeline Terto, José Vieira Silva, Cícero Almeida

**Affiliations:** 1 Laboratório de Recursos Genéticos, Campus Arapiraca, Universidade Federal de Alagoas, Arapiraca, AL, Brazil; 2 Laboratório de Fisiologia de Plantas, Universidade Federal de Alagoas, Arapiraca, AL, Brazil

**Keywords:** gene expression, plant breeding, quantitative RT-PCR

## Abstract

Drought is a major factor in decreased sugarcane productivity because of the resulting morphophysiological effects that it causes. Gene expression studies that have examined the influence of water stress in sugarcane have yielded divergent results, indicating the absence of a fixed pattern of changes in gene expression. In this work, we investigated the expression profiles of 12 genes in the leaves of a drought-tolerant genotype (RB72910) of sugarcane and compared the results with those of other studies. The genotype was subjected to 80–100% water availability (control condition) and 0–20% water availability (simulated drought). To analyze the physiological status, the SPAD index, Fv/Fm ratio, net photosynthesis (A), stomatal conductance (*g*_*s*_) and stomatal transpiration (*E*) were measured. Total RNA was extracted from leaves and the expression of SAMDC, ZmPIP2-1 protein, ZmTIP4-2 protein, WIP protein, LTP protein, histone H3, DNAj, ferredoxin I, β-tubulin, photosystem I, gene 1 and gene 2 was analyzed by quantitative real-time PCR (RT-PCR). Important differences in the expression profiles of these genes were observed when compared with other genotypes, suggesting that complex defense mechanisms are activated in response to water stress. However, there was no recognizable pattern for the changes in expression of the different proteins associated with tolerance to drought stress.

Sugarcane belongs to the genus *Saccharum* L., tribe Andropogoneae, family Poaceae. Modern commercial varieties are derived from a cross between *S. officinarum* and *S. spontaneum* that, after successive backcrossing with *S. officinarum*, resulted in cultivars with 2*n* = 100–130 chromosomes containing approximately 80% *S. officinarum*, 10% *S. spontaneum* and 10% recombinants of the two genomes (D’Hont *et al*., 1996; Grivet and Arruda, 2001).

Sugarcane is of great commercial importance since it accounts for ~65% of sugar production worldwide. In addition to sugar, ethanol derived from sugarcane provides a renewable alternative fuel that causes less pollution to the environment than oil (Carson and Botha, 2002). Modern sugarcane cultivars are highly productive in tropical regions. However, drought is a major cause of decreased productivity because of the morphophysiological effects that water deficiency causes to sugarcane, *e.g*., reduced photosynthesis and growth inhibition. These limitations have stimulated breeding programs to develop new varieties of sugarcane with greater efficiency of water use (Yordanov *et al*., 2000; Silva *et al*., 2008; Ghannoum, 2009).

The mechanisms of plant stress responses are highly important to agriculture because of their direct link to production systems. Proteins such as heat shock proteins, peroxidases and water transport proteins are involved in plant protection mechanisms under conditions of low water availability (Xiong *et al*., 2002; Casu *et al*., 2005; Wang *et al*., 2004; Borges *et al*., 2001). In addition, differential expression of some sugarcane genes associated with tolerance to water stress has been reported. Thus, Rodrigues *et al*. (2009) analyzed 3,575 ESTs in a drought-tolerant sugarcane cultivar and found 165 differentially expressed genes, indicating a large number of genes associated with drought tolerance. However, there is currently no information on the gene expression patterns in other genotypes classified as tolerant. In this study, we examined the gene expression profiles in leaves of the drought-tolerant sugarcane cultivar RB72910 under drought conditions and compared them to the expression profiles of other accessions.

The experiment was done in a greenhouse using two treatments: a control treatment and a water stress treatment with four replications in a completely randomized experimental design. Each plot consisted of a pot containing one plant. The genotype RB72910 assigned by the Sugarcane Breeding Program (PMGCA/RIDESA/UFAL) and classified as highly tolerant to drought was used for this study. To ensure greater homogeneity of seedlings, buds were pre-grown in individual boxes (40 cm long × 30 cm wide × 10 cm high) containing a substrate of coconut pulp, filter cake and black earth at a ratio of 1:1:2 (m/m), respectively, and a density of 16 buds per box. The buds were pre-treated with carbendazim fungicide at the concentration recommended by the manufacturer. Fifteen days after planting in the boxes, the best seedlings were transferred and individually planted in plastic pots (30 cm × 30 cm diameter), each one containing 12 kg of the same substrate as described above.

The humidity for the permanent wilting point (−1.5 MPa) and field capacity (−0.03 MPa) of the substrate dry mass to define treatments were 16.92 and 22.54%, respectively, obtained from a moisture retention curve of the substrate and established by gravimetric analysis. The treatments were defined as follows: moisture close to field capacity for the test without water stress, with 80–100% water availability (control) and 0–20% water availability (stressed) for testing under severe stress. All pots from both treatments were weighed daily and their masses corrected according to the available water in the substrate. The pots were kept at a humidity of 80–100%, with daily watering at 08:00 a.m. for up to 66 days of cultivation (DC) in order to replace evapotranspired water. Test measurements were taken during early crop development as this corresponded to the most sensitive period to water stress, and at 70 DC with four days of water deficiency for the stress treatment. To determine the water deficiency status, the physiological variables were analyzed at 68 and 70 days after cultivation (two and four days after water stress, respectively).

The Fv/Fm ratio was obtained by measuring chlorophyll-a fluorescence emission with a portable light-modulated fluorometer (Opti-Sciences, model OS1-FL, Hudson, USA) after the leaves had adapted to the dark (~20 min). The maximum fluorescence (Fm) was obtained with the aid of plastic clips, followed by the variable fluorescence (Fv), such that the PSII maximum quantum yield (Fv/Fm ratio) could be determined. Measurements were taken between 11 a.m. and 12 a.m. that corresponded to the most important time with respect to temperature and solar radiation for that region. This was also the period in which there was probably more damage to the photosynthetic apparatus, as shown instantly in the PSII. Two readings were taken in the middle portion of the leaf +1 for each plant.

Measurements of net photosynthesis (A), stomatal conductance (*g*_*s*_) and stomatal transpiration (*E*) were made using an IRGA infrared gas analyzer (ADC, model LCi, Hoddesdon, UK) with an air flow of 300 mL/min and a coupled light source of 995 μmol/m^2^/s, at relative humidity and room temperature. The measurements were made between 8 a.m. and 11 a.m. in the middle portion of the leaf +1, with readings taken for each plant and each repetition. The leaf chlorophyll content was estimated using the SPAD index and an SPAD-502 chlorophylometer (Minolta Corporation, Ramsey, USA), with averages obtained from eight readings for each plant. The variables were analyzed using R package software and the results expressed graphically.

Total RNA was extracted from leaves of control and drought-stressed plants using commercial Trizol reagent (Invitrogen, Carlsbad, USA), according to the manufacturer’s instructions. The samples were a mixture of leaves from the four replications of each treatment. cDNA was produced using 2 μg of total RNA as the template and a PCR-select cDNA subtraction kit, in which 2 μL of total RNA and 1 μL of cDNA synthesis primer (10 μM) were incubated for 70 °C for 2 min in a thermal cycler and then cooled on ice. After 2 min, 2 μL of 5x first-strand buffer, 1 μL of dNTP (10 mM each), 1 μL of sterile H_2_O and one microliter of AMV reverse transcriptase (20 units/μL) were added and the reaction was incubated for 90 min at 42 °C in an air incubator.

The expression profiles of 12 genes (Table 1) in sugarcane leaves (RB72910 genotype) under drought-stress and a no-stress control were analyzed by real-time RT-PCR. The sugarcane β-tubulin gene was used as an internal reference (housekeeping gene), as described by Iskandar *et al*. (2004) and Rodrigues *et al*. (2009) for relative expression of the 11 genes. The real-time PCR reactions were done using SYBR Green master mix (Fermentas) in an ABI 7500 thermocycler containing 65 ng of cDNA, 25 nM of each primer and 12.6 μL of SYBR Green master mix in a final volume of 25 μL. Amplification was done at 50 °C for 2 min, 95 °C for 10 min and 40 cycles at 95 °C for 15 s and 60 °C for 1 min. Three replicates were run and analyzed independently.

**Table 1:** Genes analyzed by quantitative real-time RT-PCR and their regulation profiles.

GenBank accession number^a^	Gene	Description^b^	Regulation profile		
			RB72910	SP83-5073^b^	SP90-1638^b^
CA222437	β-Tubulin	Housekeeping gene	-	-	-
CA127376	SAMDC	S-adenosylmethionine decarboxylase [EC 4.1.1.50] [*Zea mays*]	Down	Up	-
CA120560	Protein ZmPIP2-1	Plasma membrane integral protein ZmPIP2-1 [*Zea mays*]	Down	Up	-
CA128872	Protein ZmTIP4-2	Tonoplast membrane integral protein ZmTIP4-2 [*Zea mays*]	Up	-	Up
CA127367	Protein WIP	Wound-induced protein [*Medicago sativa* subsp. varia]	Down	Up	Up
CA119309	Protein LTP	Lipid transfer protein [*Setaria italica*]	Down	Up	Down
CA116806	Histone	Histone H3 (H3-1.1) [*Oryza sativa*]	Down	Up	Up
CA300174	DNAj	DNAj protein	Up	Down	Up
CA293774	Ferredoxin I	Ferredoxin I, chloroplast precursor (Fd I) [*Zea mays*]	Up	-	Down
CA116652	Photosystem I	Photosystem I complex PsaN subunit precursor [*Zea mays*]	Dow	-	Down
CA122935	Gene1	No match	Up	Up	Down
CA129393	Gene2	No match	Down	Up	Up

a Gene accession number in the NCBI database.

b Described as tolerant by Rodrigues *et al*. (2009).

c Described as sensitive by Rodrigues *et al*. (2009).

The sugarcane genotype used in this study has a high growth capacity in conditions of low water availability (data from PMGCA-RIBESA). The genes examined here were reported by Rodrigues *et al*. (2009) to show different levels of expression in association with tolerance to drought stress. In the present study, the physiological development of plants was assessed using the SPAD index, Fv/Fm ratio, net photosynthesis (A), stomatal conductance (gs) and stomatal gas exchange or transpiration (E). As shown in Table S1, there was a significant difference in A, gs and E when the genotype was grown under conditions of low water availability. Net photosynthesis decreased from 11 μmol CO_2_/m^2^/s in control plants to 3.6 μmol CO_2_/m^2^/s in the stress-treated plants. For gas exchange, the results were 2.09 mmol H_2_O/m^2^/s for control plants and 0.79 mmol H_2_O/m^2^/s for stressed plants, while for stomatal conductance the results were 0.11 and 0.03 mmol H_2_O/m^2^/s for control and stressed plants, respectively (Figure 1).

**Figure 1:**
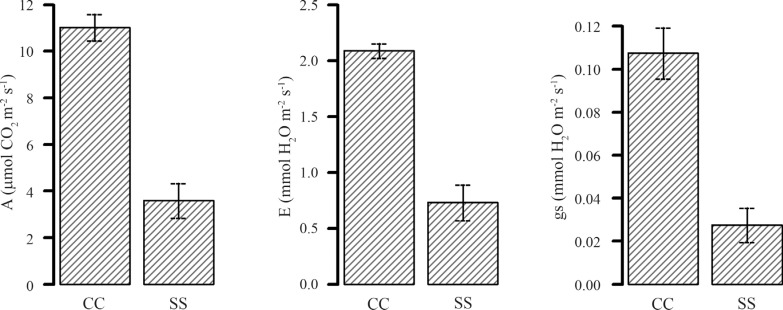
Physiological parameters of plants during water deficiency. Net photosynthesis (A), stomatal conductance (*g*_*s*_), stomatal transpiration (*E*). Error bars indicate the standard deviation (± sd).

Gene expression was assessed in triplicate using quantitative PCR. The Ct values for each gene under different experimental conditions (Figure 2) were used to obtain the relative level of gene expression by 2^−ΔΔCt^ methodology, with β-tubulin as the reference gene (Figure 3). There were no changes in the expression of gene 1, histone, photosystem I, ZmTIP4-2 and SAMDC, while DNAj, gene 2 and ferrodoxin 1 showed small alterations in expression. In contrast, when analyzed by real time RT-PCR (Figure 2) and semi-quantitative PCR (Figure S1), there were marked changes in the expression of LTP protein, WIP protein and ZmPIP2-1 (Figure 3). Under conditions of water stress, the DNAj and β-tubulin genes showed enhanced and decreased expression, respectively.

**Figure 2:**
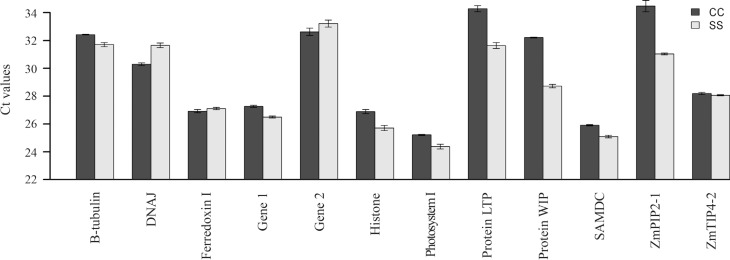
RNA transcription profiles represented as cycle threshold (Ct) values in different genes. Error bars indicate the standard deviation (± sd).

**Figure 3:**
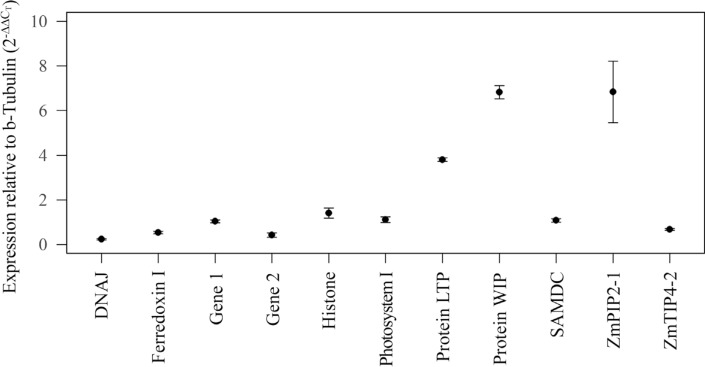
Relative gene expression analyzed by real-time PCR. Relative quantification was performed through the 2^−ΔΔCt^ method using β-tubulin as the reference gene. Error bars indicate the standard deviation (± sd).

Genomic, transcriptomic, proteomic and metabolomic studies have sought to elucidate the mechanisms of plant responses to drought stress. The responses observed in these investigations include changes in the expression of genes associated with plant maintenance, protein synthesis and turnover, and metabolic alterations (reviewed by Shanker *et al*., 2014). However, the patterns of gene expression vary among different genotypes, indicating that there are different responses associated with water stress.

The sugarcane genotype RB72910 was identified by the PMGCA/RIDESA Breeding Program as being drought-tolerant and Rodrigues *et al*. (2009,^2011^) described another genotype (SP83-5073) as tolerant to water stress. Since these two genotypes showed similar tolerance, one would expect similar profiles of gene expression in response to water stress. However, this was not the case since no stability was noted in the expression levels of the two genotypes. Comparison of the expression of 12 genes in a drought-tolerant genotype with those described by Rodrigues *et al*. (2009) indicated important differences in the expression profiles of the two genotypes (Table 1); these differences suggest the existence of divergent mechanisms for dealing with drought tolerance. Although these differences could be explained by the use of different experimental conditions, it should be noted that the experimental conditions were similar in the two studies. The major differences involved ZmPIP2-1, WIP protein, LTP protein and DNAj, as mentioned above.

A data mining analysis for sugarcane done using the Sugarcane Expressed Sequence Tags (SUCEST) database revealed an abundant expression of genes encoding chaperones, co-chaperones and other proteins linked to protection against stress in sugarcane (Borges *et al*., 2001). The genes most commonly encountered by Borges *et al*. (2001) were those responsible for the synthesis of chaperone HSP70 (heat-shock protein) and its co-factors such as HSP40, in addition to encoders of the proteins HSP90, HSP100 and small HSP chaperones (Wang *et al*., 2004). The chaperone activity of small HSPs has been associated with the heat stress response in sugarcane (Tiroli and Ramos, 2007). Gene expression profiles analyzed by microarrays in sugarcane leaves identified 165 genes in response to water stress (Rodrigues *et al*., 2009).

The enhanced expression of DNAj or HSP40 obtained by subjecting genotype RB72910 to water stress corroborated the data from *in silico* analysis showing that in conditions of limited water availability these genes were widely expressed. Based on cultivar behavior and productivity in field experiments, RB72910 is classified as tolerant to water stress in the RIDESA sugarcane breeding program database. However, DNAj showed up-regulation in sugarcane (Rodrigues *et al*., 2009) and potato (Vasquez-Robinet *et al*., 2008) genotypes, suggesting that RB72910 has a different mechanism of drought tolerance.

WIP protein is responsible for the restoration of plant tissues after attack by herbivores and abiotic agents. Enhanced expression of this protein is expected under conditions of stress, while ZmPIP2-1, which belongs to the aquaporin protein group, is expressed when the cells are in elongation. As shown here, both of these genes showed reduced expression in RB72910, in contrast to the findings of Rodrigues *et al*. (2009). According to Cheong *et al*. (2002), differences in gene expression may reflect the activation of defense mechanisms, including the reorganization of metabolism.

Plant leaves have a surface cuticle that provides a protective barrier against environmental adversities, such as drought. Lipid transfer protein (LTP) is present on leaves, but its functions are not yet known. Our results showed that LTP was down-regulated during water deficiency in RB72910; Rodrigues *et al*. (2009) reported similar findings for this genotype. The β-tubulin gene has been used as a reference gene in other studies (Iskandar *et al*., 2004; Rodrigues *et al*., 2009), but our findings indicated variability/instability in the expression of this gene.

Overall, the results of this investigation show that the patterns of gene expression vary in different genotypes classified as drought-tolerant. This variability suggests a high degree of complexity in the response of sugarcane to water stress. Studies looking at the patterns of gene expression in plants with different responses to water stress provide one approach for identifying the genes involved. However, the interpretation of these results requires caution since genotypes with morphological and physiological characteristics associated with tolerance to drought stress may exhibit distinct patterns of gene expression; such divergent expression could lead to false positive results, *i.e*., genes being incorrectly associated with tolerance to drought stress.

## Supplementary Material

The following online material is available for this article:

Figure S1 - Differential gene expression in the sugarcane genotype RB72910

Table S1 - Statistical analysis for the physiological trait

This material is available as part of the online article from http://www.scielo.br/gmb.
