# Supplementary material for: Expression profiles of sugarcane under drought conditions: Variation in gene regulation
Source: Genet Mol Biol. 2015 Oct-Dec;38(4):465–9. doi: 10.1590/S1415-475738420140288 (PMC4763319; doi:10.1590/S1415-475738420140288)
Supplement: Figure S1 - [file 1415-4757-gmb-S1415-475738420140288-s001.pdf]

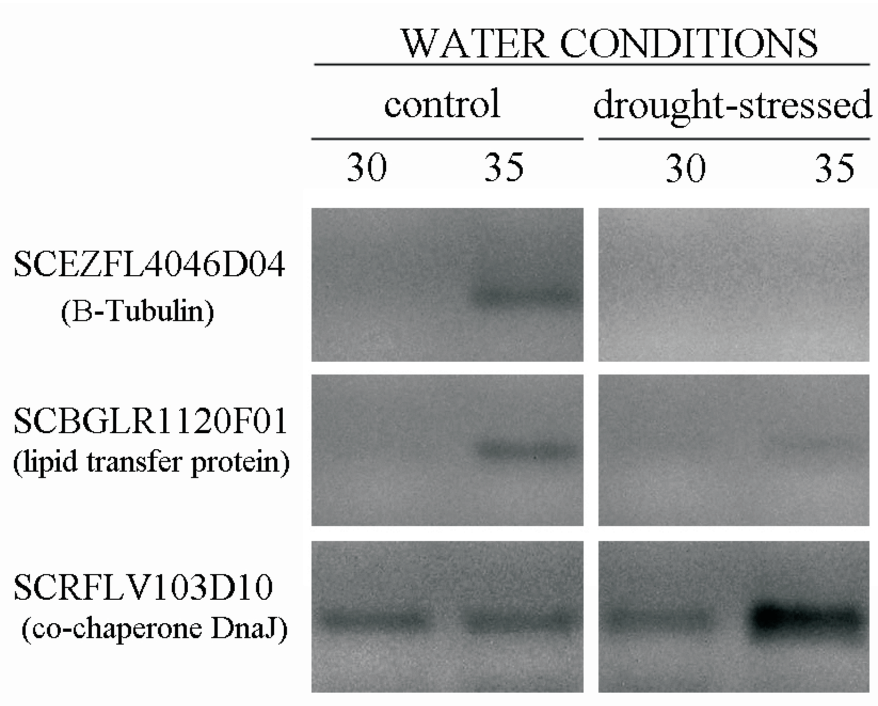

Figure S1 - Differential gene expression analyzed by RT-PCR in the sugarcane genotype RB72910 under simulated drought stress. Three genes (β-tubulin, LTP and DNAj) were amplified by PCR with 30 and 35 cycles and the amplification products were electrophoresed on 3% (w/v) agarose gels.
