# Supplementary material for: Expression profiles of sugarcane under drought conditions: Variation in gene regulation
Source: Genet Mol Biol. 2015 Oct-Dec;38(4):465–9. doi: 10.1590/S1415-475738420140288 (PMC4763319; doi:10.1590/S1415-475738420140288)
Supplement: Table S1 - [file 1415-4757-gmb-S1415-475738420140288-s002.pdf]

**Table S1 -** Analysis of variance and coefficient of variance for the physiological traits SPAD index, Fv/Fm ratio, net photosynthesis (A), stomatal transpiration (E) and stomatal conductance (gs).

| Source of variance | df | Mean Sq | | | | |
|---|---|---|---|---|---|---|
| | | SPAD index | Fv/Fm | A | E | gs |
| Treatments | 1 | 2.7 | 0.0016 | 220.9* | 7.38* | 0.026* |
| Days | 1 | 75.7 | 0.0009 | 0.51 | 0.01 | 0.001 |
| Treatments x Days | 1 | 3.4 | 0.0006 | 0.0008 | 0.01 | 0.002 |
| Residuals | 12 | 31.2 | 0.0004 | 4 | 0.14 | 0.008 |
| CV% | - | 15 | 2.6 | 27 | 26 | 43 |

*p<0.05
